# Generation of intense and coherent sub-femtosecond X-ray pulses in electron storage rings

**DOI:** 10.1038/s41598-020-67027-0

**Published:** 2020-06-22

**Authors:** J.-G. Hwang, G. Schiwietz, M. Abo-Bakr, T. Atkinson, M. Ries, P. Goslawski, G. Klemz, R. Müller, A. Schälicke, A. Jankowiak

**Affiliations:** Helmholtz-Zentrum Berlin (HZB), Albert-Einstein Straße 15, Berlin, 12489 Germany

**Keywords:** Particle physics, Plasma physics, Statistical physics, thermodynamics and nonlinear dynamics

## Abstract

Temporally short X-ray pulses are an indispensable tool for the study of electron transitions close to the Fermi energy and structural changes in molecules undergoing chemical reactions which take place on a time-scale of hundreds of femtoseconds. The time resolution of experiments at 3^*rd*^ generation light sources which produce intense synchrotron radiation is limited fundamentally by the electron-bunch length in the range of tens of picoseconds. Here we propose a new scheme for the generation of intense and coherent sub-femtoseconds soft X-ray pulses in storage rings by applying the Echo-Enabled Harmonic Generation (EEHG) method. Many issues for obtaining the EEHG structure such as two modulators and a radiator are solved by a paradigm shift in an achromatic storage ring cell. Numerical demonstration of the feasibility of the scheme for the BESSY II beam parameters is presented.

## Introduction

Third generation synchrotron radiation facilities have played important roles in the progress of multiple scientific fields by producing intensive X-ray pulses to study the structure and function of matter. The time resolution of experiments is limited fundamentally in time resolution by the electron-bunch length of 30 to 100 ps full width at half-maximum (FWHM). Fast reactions in the sub-picosecond range, on which most chemical reactions and some phase transitions take place, have been accessible only in the visible regime by lasers^1,2^. During the past decade, this science motivated the development of future light sources based on linear accelerators, e.g., X-ray free electron lasers and energy recovery linacs. This requires a high quality electron beam with a short length to produce ultrashort and intense X-ray pulses^3–8^. Contrary to the construction of new light sources, techniques for the manipulation of the time structure of relativistic electron bunches stored in a storage ring have been devised^9–13^, experimentally demonstrated^14–17^, and utilized to X-ray absorption spectroscopy^18^ up to a few MHz repetition rate. The femtoslicing facility at BESSY II provides 100 fs long X-ray pulses with 6 kHz repetition rate. Ultrafast structural dynamics associated with phase transitions in solids, chemical reactions, and rapid biological processes^19^ are commonly investigated. However, the average photon flux of the sliced pulses is fundamentally limited to 10^5^–10^6^ photons/s/0.1% BW.

We suggest a novel scheme for the generation of intense, temporally coherent, sub-femtosecond X-ray pulses in storage rings by applying an echo-enabled harmonic generation (EEHG) method^20,21^. Particularly, the proposed scheme is compatible with storage ring based synchrotron sources, which typically have limited construction space in straight sections. This method makes use of one achromatic cell of the ring as a dispersive section for the density manipulation (partial bunch compression) of an electron bunch. To obtain the EEHG structure two modulators and a radiator are needed. This method exploits the limited space of existing storage rings by connecting two straight sections. We demonstrate the feasibility of the generation of 0.83 keV soft X-ray pulses of about 0.29 fs FWHM with a photon flux of 1.0 × 10^9^ photons/s/0.1% BW and rms bandwidth of 0.02 nm in accordance with the repetition rate of 6 kHz. In the scheme proposed by C. Evain^22^, by means of two additional chicanes, the R51 element can be exactly tuned to zero, mandatory to maintain the high bunching factor. In contrast, in our scheme these chicanes are not needed. Therefore, the dilution of the micro bunching structure by quantum fluctuation is minimized. We also include the numerical illustration of the feasibility of the scheme. In the numerical study, the effects of high-order aberration are evaluated with high precision because these effects can degrade the bunching efficiency. In addition, we studied a concept for the compensation of the magnetic aberrations by adjusting the strength of chromatic sextupole magnets in the achromatic cell.

Many free electron laser (FEL) seeding techniques using laser-assisted electron-beam manipulation schemes designed to produce a beam-density modulation for enhancing the temporal coherence of synchrotron radiation at high harmonics have been proposed and demonstrated experimentally^23–27^. These techniques, however, are not suitable for most storage ring-based 3^*rd*^ generation light sources due to limited space and technical constraints. Storage rings have conventionally straight sections of 5 to 7 m in length between two dispersive sections, required essentially for the maximum use of undulators to generate intense and temporally coherent radiation from electron beams.

**Figure 1:**
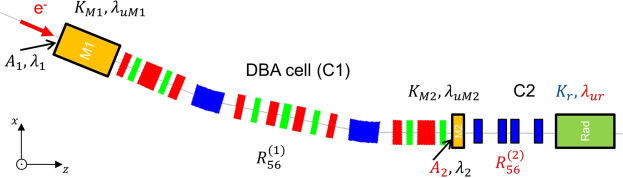
Schematic view of the proposed scheme by connecting two straight sections using an achromatic cell as a dispersive magnetic chicane. For BESSY II, the first magnetic chicane *C*1 is a double bend achromatic (DBA) cell and the first modulator *M*1 is installed inside the first straight section. The second modulator *M*2, magnetic chicane *C*2, and the radiator are installed at the second straight section.

Here, we propose a new scheme that utilizes a FEL seeding technique for storage rings. Among various FEL seeding techniques, we focus on the short-pulse generation scheme using an EEHG-FEL method proposed by A. Zholennts and G. Penn^28^. This scheme can generate an intense short pulse with relatively short-length modulators and radiator. The scheme is essentially based on a two-step manipulation of an energy modulation produced by the interaction of an electron bunch with two laser pulses. Quantitatively, each laser involves a specific wavelength ${\lambda }_{1}$ (and ${\lambda }_{2}$) inside a wiggler magnet *M*1 (and *M*2) and leads to a corresponding relative momentum deviation Δ*p*/*p*. Once in a magnetic chicane, this leads to a path-length variation Δ*L* that is described by the so-called transport matrix element *R*_56_ = Δ*L*/(Δ*p*/*p*). Since storage rings have typically a momentum compaction factor in the order of 10^−3^ with the circumference of hundreds of meters, the local momentum compaction factor of one achromatic cell is sacrificed to produce a characteristic electron distribution in the longitudinal phase-space. Here narrow bands of electrons are interleaved within the empty phase-space. We thus can apply the FEL seeding scheme which consists of two modulators with seeding lasers, two dispersive magnetic chicanes with matrix elements ${R}_{56}^{(1)}$ and ${R}_{56}^{(2)}$, and one radiator into two straight sections. The schematic layout of the proposed scheme in a storage ring is shown in Fig. 1.

The DBA cell involving ${R}_{56}^{(1)}$ used as first magnetic chicane in our scheme is predetermined by the lattice design of the storage ring. Parameters such as the wavelength of lasers *λ*_1_ and *λ*_2_, the period and undulator parameter of first and second modulators ${\lambda }_{uM1}$, ${\lambda }_{uM2}$, ${K}_{M1}$ and ${K}_{M2}$ which are associated with the wavelength of the lasers, and the period of radiator ${\lambda }_{ur}$ can be optimized in the design phase to achieve a high up-conversion efficiency of the harmonic generation process for the echo pulse generation. The modulator periods, laser wavelengths, and R${\,}_{56}^{(1)}$ are in practice not adjustable after the installation of the devices. The undulator parameter is $K=eB{\lambda }_{u}/2\pi {m}_{e}c$, where *B* is the peak magnetic field, *e* and *m*_*e*_ are the electron charge and mass, *λ*_*u*_ is the undulator period, and *c* is the speed of light. The microbunching with a small period *λ*_*r*_, corresponding to a harmonic number $h={\lambda }_{2}/{\lambda }_{r}=|n+{\lambda }_{2}/{\lambda }_{1}|$ is achieved when^20,28^:

1 $$\Delta {E}_{2}\simeq \frac{E}{|{R}_{56}^{(1)}|}\frac{{\lambda }_{1}}{2\pi }(|n|+0.809\,|n{|}^{1/3}),$$

2 $${R}_{56}^{(2)}=-\,\frac{2\pi {R}_{56}^{(1)}{\lambda }_{2}-{\lambda }_{1}{\lambda }_{2}\frac{E}{{\sigma }_{E}}}{2\pi ({\lambda }_{1}n+{\lambda }_{2})},$$

where *E* is the electron-beam energy, Δ*E*_2_ is the amplitude of energy modulation produced at the second modulator, *λ*_1_ and *λ*_2_ are the wavelength of the lasers at the first and second modulator, respectively, *n* is a large integer number, and *σ*_*E*_ is the energy spread of the bunch. This method requires a well-defined energy modulation produced by the combination of a laser and wiggler magnet^29^.

**Figure 2:**
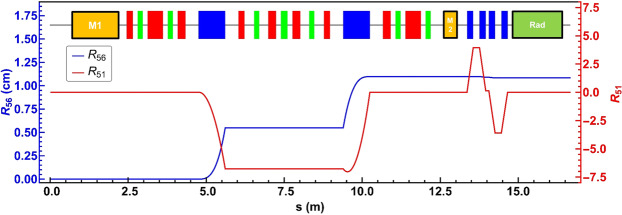
The evolution of ${R}_{56}$ and ${R}_{51}$ elements along the beamline of the BESSY II storage ring. The beam line has the ${R}_{56}^{(1)}$ of about 11 mm which is suitable for the EEHG scheme. Yellow, red, green boxes represent dipole, quadrupole, sextupole magnets, respectively.

To verify the feasibility of the scheme described above, we demonstrate the generation of an intense and short X-ray pulse with a carrier frequency at the nitrogen K-edge, using electron beam parameters of BESSY II^30,31^. The parameters of the femtoslicing bunches with rms bunch length of 30 ps and beam current of 4 mA are used in further calculation. First, in order to confirm the generation of the microbunching structure, the evolution of ${R}_{56}^{(1)}$ in the DBA cell of the BESSY II storage ring is calculated by using the Elegant code^32^. The lattice functions are established based on linear optics from closed orbit measurements of the present BESSY II lattice which was locally modified to implement the Femtoslicing and EMIL beamlines^2,33,34^. The momentum compaction ${\alpha }_{c}$, circumference *C* and superperiod *S* of the storage ring are 7.3 × 10^−4^, 240 m, and 16, respectively^30,31^. Therefore, ${R}_{56}^{(1)}$ of a single DBA cell is expected to be ${\alpha }_{c}\times C/S=11\,{\rm{mm}}$, in accordance with the numerical calculation. This is suitable for generating a microbunching structure with a small period *λ*_*r*_. ${R}_{56}^{(1)}$ however, is not an adjustable parameter because the lattice functions of a DBA cell should keep the achromatic condition. The lattice design defines fundamentally the operation of the storage ring. The evolution of *R*_51_ and *R*_56_ along the beamline are calculated numerically and shown in Fig. 2.

The proposed scheme achieves the flexibility of the radiation wavelength at the radiator by adjusting the amplitude of the energy modulation at the second modulator Δ*E*_2_ and ${R}_{56}^{(2)}$. The optimum values of ${R}_{56}^{(2)}$ and *A*_2_ = Δ${E}_{2}/{\sigma }_{E}$ as a function of the photon energy at the radiator can be determined by Eqs. (1) and (2). In addition, the parameters of the wiggler magnet for *M*1 and *M*2 were optimized to have a period of 13.9 cm and ${K}_{M1}={K}_{M2}=15.9$ based on the existing wigglers at BESSY II. The detailed parameters are listed in Table 1.

**Table 1:** Major parameters for the generation of sub-femtosecond X-ray (0.83 keV) pulses in the BESSY II storage ring.

Parameters	Units	Value
Beam Energy *E*	GeV	1.7
Relative energy spread *σ*_*E*_/*E*		7 × 10^−4^
Bunch current	mA	4
Bunch length rms	ps	30
Modulator period length *λ*_*μM*1_ = *λ*_*μM*2_	cm	13.9
First modulator period		10
Second modulator period		1
Laser wavelength *λ*_1_ = *λ*_2_	nm	800
First laser pulse energy	*μ*J	110
First laser pulse duration FWHM	fs	200
Second laser pulse energy	*μ*J	196
Second laser pulse duration FWHM	fs	5
Radiator period length	cm	1.7
Radiator period		88
DBA cell ${R}_{56}^{(1)}$	mm	11
Magnetic chicane ${R}_{56}^{(2)}$	*μ*m	−20.7

In a further calculation, we simulated the laser-beam interaction assuming that the cross-section of the laser light in two modulators is several times larger than the transverse profiles of the electron bunch. Therefore, the energy change of an electron induced by the interaction with laser light in a wiggler is equal for all electrons at the same longitudinal location along the bunch according to the phase of the laser light at the beginning of the interaction^28^. The calculation of the energy modulation within an electron bunch was performed while the wiggler parameter of two modulators is tuned to satisfy the FEL resonance condition ${\lambda }_{1}={\lambda }_{uM1}(1+{K}_{M1}^{2})/(2{\gamma }^{2})$ where $\gamma =E/{m}_{0}{c}^{2}$. A laser pulse energy of 110 *μ*J at a wavelength of *λ*_1_ = 800 nm and duration of minimum 300 fs FWHM is required for generating an energy modulation of 2*σ*_*E*_ with the undulator periods of 10, which corresponds to about 1% of the undisturbed bunch length. The energy modulation is then transformed into a microbunching structure via the ${R}_{56}^{(1)}$ in the DBA cell. This method has the advantage of utilizing an existing storage ring (here we focus on BESSY II) with a relatively long dipole magnet at a fixed angle in C1. This magnet reduces the rms energy spread induced by quantum fluctuations of synchrotron radiation.

**Figure 3:**
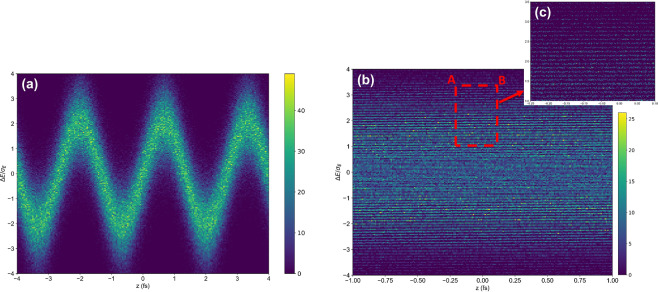
A fragment of the electron bunch in longitudinal phase space after first modulator *M*1 (**a**) and after the DBA cell *C*1 (**b**). The horizontal axis is the distance along the bunch and the vertical axis is energy deviation from the equilibrium energy normalized to the rms energy spread in the undisturbed electron bunch.

The dilution of the microbunching bandwidth due to the first dipole magnet (approximately 2.96 keV rms) is small compared to the distance between two adjacent electron-energy bands estimated to be $E{\lambda }_{1}/2{R}_{56}^{(1)}=61.8\,{\rm{keV}}$ (see Fig. 3c). These bands relate to small slices of the initial energy modulation (Fig. 3a) for a large number of laser oscillation cycles. We then reproduce the energy spread growth and spectral distributions using Elegant code. In the numerical simulation, a particle tracking simulation with 2 M macro-particles are performed over the DBA beamline with a integration step size of about 25 mm. The simulated distribution downstream of the first modulator and downstream of the DBA cell are shown in Fig. 3.

Since the particle delay produced by the combined effects of laser-induced energy spread and *R*_56_ is much larger than the wavelength of the laser, i.e. ${R}_{56}^{(1)}$ × Δ*E*_1_/$E\gg {\lambda }_{1}$, the particle density in the temporal axis is transformed into a nearly uniform time distribution (see Fig. 3b). The generation of subsequent microbunching at wavelengths much shorter than the first laser wavelength via the transformation of narrow spacing along the energy axis into short microbunches along the longitudinal coordinate is a critical step. These microbunches stand upright by the manipulation of longitudinal phase space through the second chicane ${R}_{56}^{(2)}$ to generate short pulses with a high bunching factor at the target wavelength *λ*_*r*_. The bunching factor ${b}_{k}^{h}$ quantifies the longitudinal, periodic density modulation of the electrons with respect to the radiation wavelength *λ*_*r*_; ${b}_{k}^{h}=\frac{1}{{n}_{k}}\,{\sum }_{j=1}^{{n}_{k}}\,{e}^{2\pi i{z}_{j}/{\lambda }_{r}h}$, where ${z}_{j}$ is the longitudinal position of *j*-th particle, and *n*_*k*_ is the number of particles in the *k*-th slice. The slippage effect on energy modulation in the second wiggler is carefully evaluated and the single period wiggler is adopted for this scheme^35^. With the second wiggler with one period, a laser pulse power of 196 *μ*J at *λ*_2_ = 800 nm and the duration of 5.0 fs FWHM is required for generating the energy modulation of 8.6*σ*_*E*_. It is crucial to keep the duration of the second laser as short as possible in order to suppress the radiation from the side pulses due to the multiple cycles of the laser. The generation of a few-cycle optical pulse with a pulse power of several hundreds *μ*J at the wavelength of 800 nm is experimentally proven^36,37^.

For the harmonic number *h* = 530 *λ*_*r*_ = 1.49 nm, the optimum ${R}_{56}^{(2)}$ calculated from Eq. (2) is −20.7 *μ*m and it is easily achievable with a four-dipole-magnet chicane of large curvatures which would reduce the rms energy spread induced by quantum fluctuation of synchrotron radiation. ${R}_{56}^{(2)}$ should be tunable to adjust the wavelength at the radiation. The decay length of the bunching factor due to Coulomb collisions^38^ at high harmonics is negligible since the BESSY II storage ring has a much lower peak current and a larger emittance compared to FELs.

**Figure 4:**
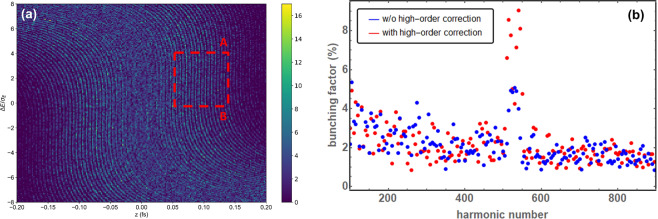
Longitudinal phase space after the second chicane *C*2 showing the microstructure inside the central peak (**a**) and the bunching factor (**b**) with and without the high-order aberration correction via sextupoles. One observes the increase of the background moving towards smaller harmonic numbers due to the bandwidth dilution at lower frequencies.

The energy is not significantly changed in the DBA cell, but the particles are delayed by the position and angle of the electrons according to *z*_1_ = *z*_0_ + *R*_56_Δ*E*/$E+{T}_{511}{x}_{0}^{2}+{T}_{512}{x}_{0}{x{\prime} }_{0}+\cdots$ with the bunch head at $z > 0$. Chromatic sextupoles were used to reduce the critical second oder terms *T*_511_ and *T*_521_ to increase the bunching factor^39,40^. After the compensation, the *T*_511_ and *T*_521_ values are reduced to be 0.32 1/*m* and 0.13, respectively, and the bunching factor is increased from 5% to about 9%. A fragment of the longitudinal phase-space showing the microbunching structure inside the central peak and the bunching factor with and without the high-order aberration correction are shown in Fig. 4.

The detuning of the local sextupole magnets, however, is not only lowering the symmetry of the lattice but also leads to a change of the chromaticity which can potentially cause instabilities and the reduction of dynamic aperture. However, these effects can be suppressed by using both chromatic and harmonic sextupole families. The modern third-generation light sources have adequate harmonic sextupole families to compensate nonlinear effects^41,42^. By the numerical simulation, we confirm the recovery of the dynamic aperture for on-momentum and off-momentum particles with a ±2% momentum deviation by correcting the chromaticity. The electron beam with its microbunching structures is transported to the 1.5 m radiator with 53 periods, a period length of 17 mm, and *K* = 1.00 tuned for the FEL resonance at a wavelength of 1.49 nm. We adopt radiator parameters based on an existing technology which is developed to cover the energy range from 0.7 to 10 keV in the EMIL beamline^43^.

**Figure 5:**
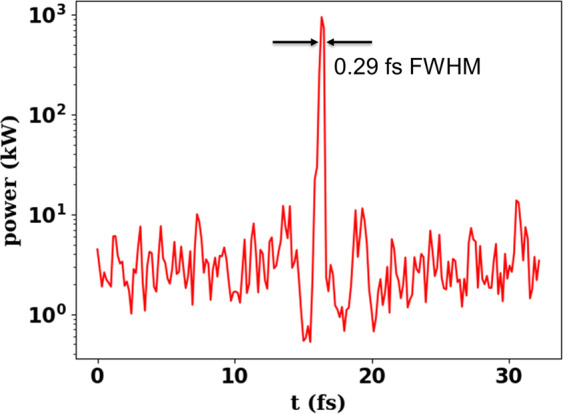
Radiation power along bunch position calculated (see Fig. 4) using GENESIS.

Figure 5 shows the calculations carried out using GENESIS code^44^ with an initial particle distribution taken from BESSY II. The peak power is 1051 kW at the carrier energy of 0.83 keV and the pulse duration of 0.29 fs FWHM. With the repetition rate of 6 kHz, the average photon flux of the pulse is 1.0 × 10^9^ photons/s/0.1% BW with rms bandwidth of 0.02 nm. This scheme provides more than two orders of magnitude higher pulse power than the present femtoslicing technique^2,19^ and reduces the X-ray pulse length below a femosecond. A bunch separation technique such as mechanical X-ray chopper^45^, PSB method^46^ and TRIBs^47^ is fundamentally necessary for suppressing the radiation from the rest of bunches. Within the EEHG bunch, the short-pulse radiation from the microbunches, however, will be superimposed on the incoherent radiation of the whole beam. This is a broad spectrum since the length of the radiator is substantially shorter than the FEL saturation length. Thus we can enhance the contrast ratio by tuning a monochromator which is often used to select a wavelength of the radiation^48^. an upgrade project of existing storage rings such as BESSY VSR^49^ generating a few high peak-current bunches can enhance the radiation power exceedingly since the radiation power of the EEHG scheme is proportional to the square of the peak current. It is possible to improve the contrast ratio by controlling angular acceptance at beamlines in conjunction with emittance blow-up by incoherent excitation such as pulse-picking by resonance excitation^50,51^. In addition, a robust speckle-free discrimination between different degrees of coherence using the optical response of metallic metasurfaces is also feasible^52^. Thee are sophisticated experimental methodologies that can resolve phenomena by the cohererent radiation^53–55^.

In order to confirm a tuning of the fundamental wavelength of emission by changing the strength of laser power *A*_2_ and ${R}_{56}^{(2)}$, the numerical calculation was performed. The optimum parameters of *A*_2_ and ${R}_{56}^{(2)}$, and results are summarized in Table 2. The tunable range of the scheme is limited by the maximum field strength of the radiator which determines a lower boundary of the photon energy and the energy spread of the laser modulated bunch which causes beam loss. For BESSY II, it was confirmed by the experiment that the acceptable energy spread is about 0.9% which corresponds to the photon energy of 830 eV.

**Table 2:** The peak power and pulse length of the EEHG scheme at different wavelengths within the tunable range.

Photon energy (eV)	optimum *A*_2_	optimum ${R}_{56}^{(2)}$ (*μ*m)	Pulse duration FWHM (fs)	Peak power (kW)
400	4.4	−42.9	0.69	1025
500	5.4	−34.2	0.49	989
600	6.5	−28.4	0.47	810
700	7.6	−24.5	0.31	771
830	8.6	−21.5	0.29	1051

In addition to the short-pulse generation scheme, it is also possible to adopt a single-stage EEHG scheme at high harmonics to directly generate an intense X-ray radiation because the modulator and radiator cover a large frequency range. Long seed laser pulses can be adopted to fully overlap the electron bunch, which results in a much higher output pulse energy and much narrower output bandwidth.

We present an analytical and numerical illustration for a novel scheme to generate intense, temporally coherent, and short sub-femtosecond X-ray pulses in storage rings by applying an EEHG method. By adopting one achromatic cell of the ring to act like a dispersive magnetic chicane used for the density manipulation of an electron bunch, a compact EEHG structure for storage ring is attainable. Two modulators and a radiator in the same straight section is no longer necessary. The proposed scheme is largely immune to static errors in laser phase and amplitude, and magnet power supplies which could cause a distortion in the lattice functions. The most critical aspect towards stable operation involves a stable carrier-envelope phase for the 5-fs laser pulse.
